# Mutated circulating tumor DNA as a liquid biopsy in lung cancer detection and treatment

**DOI:** 10.1002/1878-0261.12983

**Published:** 2021-05-26

**Authors:** Martyna Filipska, Rafael Rosell

**Affiliations:** ^1^ Germans Trias i Pujol Research Institute and Hospital Badalona Spain; ^2^ Autonomous University of Barcelona Cerdanyola del Valles Spain

**Keywords:** cancer detection, cfDNA, ctDNA, liquid biopsy, LUAD, NSCLC, TMB

## Abstract

Over the past decade, substantial developments have been made in the detection of circulating tumor DNA (ctDNA)—cell‐free DNA (cfDNA) fragments released into the circulation from tumor cells and displaying the genetic alterations of those cells. As such, ctDNA detected in liquid biopsies serves as a powerful tool for cancer patient stratification, therapy guidance, detection of resistance, and relapse monitoring. In this Review, we describe lung cancer diagnosis and monitoring strategies using ctDNA detection technologies and compile recent evidence regarding lung cancer‐related mutation detection in liquid biopsy. We focus not only on epidermal growth factor receptor (*EGFR*) alterations, but also on significant co‐mutations that shed more light on novel ctDNA‐based liquid biopsy applications. Finally, we discuss future perspectives of early‐cancer detection and clonal hematopoiesis filtering strategies, with possible inclusion of microbiome‐driven liquid biopsy.

## Graphical abstract

Liquid biopsy can greatly contribute to better detection and management of lung cancer. ctDNA detection in liquid biopsies is a powerful tool for early detection, cancer patient stratification, therapy guidance, detection of resistance, and relapse monitoring. We describe lung cancer diagnosis and monitoring strategies using ctDNA detection technologies and compile recent evidence on the identification of lung cancer‐related mutations through liquid biopsy.

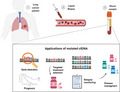

**Abbreviations**
AGR2: anterior gradient 2
AKT1: AKT serine/threonine kinase 1
ALK: ALK receptor tyrosine kinase
ASXL1: ASXL transcriptional regulator 1
BRAF: B‐Raf proto‐oncogene
CAPP‐Seq: cancer profiling by deep sequencing
CDK4: cyclin‐dependent kinase 4
cfDNA: cell‐free DNA
cffDNA: cell‐free fetal DNA
CH: clonal hematopoiesis
CNV: copy number variation
CTC: circulating tumor cell
ctDNA: circulating tumor DNA
cTMB: corrected tumor mutation burden
CTNNB1: catenin beta 1
DNMT3A: DNA methyltransferase 3 alpha
ds: double‐stranded
EGFR: epidermal growth factor receptor
ERBB2: erb‐b2 receptor tyrosine kinase 2
FGF: fibroblastic growth factor
FOXA1: forkhead box A1
JAK: Janus kinase
KEAP1: kelch‐like ECH‐associated protein 1
KRAS: KRAS proto‐oncogene GTPase
LCMC: Lung Cancer Mutation Consortium
LSCC: lung squamous cell carcinoma
LUAD: lung adenocarcinoma
mbDNA: microbial DNA
MDM2: MDM2 proto‐oncogene
MEK1: serine/threonine protein kinase MEK1
MET: MET proto‐oncogene
mtDNA: mitochondrial DNA
NGS: next‐generation sequencing
NKX2‐1: NK2 homeobox 1
NRAS: NRAS proto‐oncogene GTPase
NSCLC: non‐small‐cell lung cancer
OS: overall survival
PFS: progression‐free survival
PIK3CA: phosphatidylinositol‐4,5‐bisphosphate 3‐kinase catalytic subunit alpha
PTEN: phosphatase and tensin homolog
RB1: RB transcriptional corepressor 1
RET: ret proto‐oncogene
ROS1: ROS proto‐oncogene 1
RTK: receptor tyrosine kinase
SEER: Surveillance, Epidemiology and End Results
SMARCA4: SWI/SNF‐related, matrix‐associated, actin‐dependent regulator of chromatin, subfamily a, member 4
ss: single‐stranded
ST7: suppression of tumorigenicity 7
STK11: serine/threonine kinase 11
TCGA: The Cancer Genome Atlas
TET2: tet methylcytosine dioxygenase 2
TGFA: transforming growth factor alpha
TKI: tyrosine kinase inhibitor
TMB: tumor mutation burden
TP53: tumor protein p53
TTF‐1: transcription termination factor 1

## 1 Introduction

Personalized medicine—specifically precision oncology—nowadays provides molecular characterization of a patient’s tumor via tissue biopsy and can help guide treatment decisions. However, to fully implement personalization in the field of oncology, it is necessary to have an easily accessible and less invasive way to determine and follow the molecular makeup of a tumor from the moment of its detection and over the treatment of the disease. One such approach is through a liquid biopsy, where the genetic characterization of the tumor can be assessed through a biofluid sample. The term ‘liquid biopsy’ refers to tumor‐derived analytes sampled from various biological fluids, usually blood, but also other clinical specimens, such as urine, saliva, ascites, and cerebrospinal fluid [1].

The constant development of technologies to detect cell‐free DNA (cfDNA) with high sensitivity has facilitated the employment of liquid biopsies in diverse clinical applications, including in oncology. Analysis of circulating tumor DNA (ctDNA) obtained from plasma at multiple time points throughout the course of the disease allows for patient stratification for treatment (known as ‘companion diagnostics’), screening, monitoring response to the selected treatment, and detection of minimal residual disease after surgery.

In this article, we define the terms of cfDNA and ctDNA, describe their properties, and outline the historical breakthroughs in ctDNA detection. Most importantly, we then aim at summarizing the most recent state‐of‐the‐art developments in ctDNA utilization in diagnosis, including early‐stage detection, treatment selection, and follow‐up, in non‐small‐cell lung cancer (NSCLC) patients.

## 2 cfDNA and ctDNA: history, definitions, and properties

cfDNA refers to degraded DNA fragments of usually 167 bp length that are released into the blood [2, 3]. The specific length may result from the action of a caspase‐dependent endonuclease that cleaves DNA after a core histone and its linker. Recent studies have shown a distinct nuclear fragmentation pattern, with variable fragment lengths of cfDNA, from different tissue of origin [4, 5, 6, 7]. Several different types of cfDNA have been described in the circulation, including both double‐stranded (ds) and single‐stranded (ss) DNA particles. In humans, cfDNA originates from all cells, yet the vast majority is known to be of hematopoietic provenance [8, 9, 10, 11]. Within the ‘classical’ cfDNA, we can distinguish more specific subclasses based on its site of origin or mechanism of release, for example, mitochondrial DNA (mtDNA) [12], cell‐free fetal DNA (cffDNA) [13], extrachromosomal circular DNA [14], as well as microbial DNA (mbDNA) [15, 16, 17].

The first record of cfDNA detection in blood serum and plasma was in 1948, by Mandel and colleagues [18]. Later, in 1977, higher levels of cfDNA were detected in patients with pancreatic cancer compared with healthy controls [19], which led to the hypothesis that tumors release DNA fragments to the circulation. In 1983, Shapiro and colleagues confirmed correlations between benign vs. malignant tumors and cfDNA concentration [20]. Later, Stroun *et al*. [21] demonstrated that some of these DNA fragments were of tumor origin, due to their genomic instability. In the early 1990s, two independent studies noted the presence of specific KRAS proto‐oncogene GTPase (*KRAS*) and NRAS proto‐oncogene GTPase (*NRAS*) mutations in cfDNA from pancreatic adenocarcinoma [22] and patients with acute myelogenous leukemia [22, 23]. This fraction of cfDNA was later termed as ‘circulating tumor DNA’ (ctDNA).

ctDNA refers to cfDNA fragments that are released into the bloodstream from primary tumor or metastatic cells and display tumor‐specific point mutations, chromosomal rearrangements, copy‐number variation (CNV), and DNA methylation [24, 25, 26]. Importantly, ctDNA is more fragmented than cfDNA, resulting in a much higher <100 bp fraction in the plasma [27]. The 10‐bp periodicity observed for fragments smaller than 167 bp [3, 28] corresponds to a turn of the DNA helix wrapped around the histone. This might protect one part of the DNA from the nucleases present in the blood. This specific fragmentation pattern suggests that apoptosis may be a major source of cfDNA and that histones may be the key protein complex associated with DNA in the blood. The release of longer ctDNA fragments from tumor cells has been associated with necrotic cell death and occurs via active processes in living cells [11, 29, 30]. ctDNA fragments that are released into the circulation mirror the tumor status, its evolution, and the genomic alterations present in primary and/or metastatic tumors [11].

In a pan‐cancer study involving 640 patients, Bettegowda *et al*. [31] demonstrated that ctDNA analysis might allow monitoring of the therapeutic response, tracking resistance, and, in some cases, early detection of localized malignancies. They have also shown a correlation between tumor burden and stage of the disease. Significant differences in ctDNA levels were seen between cancer types, and the median cfDNA concentration was shown to be 100‐fold higher in patients with stage IV versus stage I disease [31].

ctDNA has a notably short half‐life in the bloodstream [32], and this characteristic is an important feature in analyzing dynamics of the mutations and tumor burden after surgery or systemic treatment throughout the disease. Thus, real‐time tumor dynamics might be monitored through ctDNA analysis for early prediction and assessment of drug response, as well as early intervention independent of detection by imaging examinations or clinical symptoms [26]. A recent study developed a mathematical model to predict the shedding rate of early‐stage NSCLC [33]. From this study, it has been estimated that there would be an average of only 1.7 genome copies of ctDNA in 15 mL of blood for lung tumors with a volume of 1 cm^3^.

In general, detection of ctDNA requires the presence of typical mutations that can be readily detected by simple sequencing techniques, proving the presence of tumor. With the advent of genomic information from the most recent cancer genome sequencing studies, it has become clear that practically all cancers of every type harbor somatic alterations. Cancer somatic mutations occur at minor frequencies in normal cell populations and therefore provide impeccably specific biomarkers from a biological perspective [34]. Some historic studies provide temporal analyses of the total tumor burden, as well as identifying specific mutations that appear during therapy [29, 35, 36, 37, 38, 39, 40].

Identification of somatic mutations within white blood cells might be a recurring source of discordance between tumor and total cfDNA genotyping. This phenomenon is called clonal hematopoiesis (CH) and is an aging‐related phenomenon whereby nonmalignant hematopoietic stem and progenitor cells acquire somatic alterations that can confer a selective advantage [41]. CH mutations are similar to the mutations detected in plasma and may involve both canonical CH genes, such as DNA methyltransferase 3 alpha (*DNMT3A*); tet methylcytosine dioxygenase 2 (*TET2*); ASXL transcriptional regulator 1 (*ASXL1*); and Janus kinase (*JAK*), and driver mutations related to tumors, such as *KRAS*, phosphatidylinositol‐4,5‐bisphosphate 3‐kinase catalytic subunit alpha (*PIK3CA*), and epidermal growth factor receptor (*EGFR*) mutations [42, 43, 44, 45]. Because hematopoietic cells are the primary source of cfDNA [46] and contribute somatic variants to the cfDNA pool [43, 44], several approaches to distinguish mutations derived from CH from their tumor‐derived counterparts have been proposed [42, 44, 47]. Studies that indicated that CH and tumor cfDNA have fragments of a different size distribution might also help to distinguish between the two [48, 49, 50]. Chan *et al*. [51] elaborate elegantly on further clinical implications of the CH phenomenon.

In addition to considering CH before implementing ctDNA analysis in the clinic, other strict guidelines and standard operating procedures need to be formulated. Factors like preanalytical standardization need to be well optimized. For instance, the CANCER‐ID consortium was funded between public and private sector units with the aim of establishing standard protocols for and clinical validation of ctDNA‐ and circulating tumor cell (CTC)‐based biomarkers. Lampignano *et al*. [52] compare the preanalytical and analytical workflows of cfDNA‐based techniques, and Grölz *et al*. [53] explain the importance of preserving whole‐blood specimens after blood drawn for use as liquid biopsies, and summarize preservation solutions that are currently available. Through entities like CANCER‐ID or the International Liquid Biopsy Standardization Alliance (ILSA) [54], the importance of working toward the global use of liquid biopsy in oncology practice is being well recognized.

## 3 A glimpse at lung cancer

Lung cancer remains the most common cause of cancer death worldwide, with an estimated 1.8 million deaths each year [55]. About 85% of patients are histologically grouped as NSCLC, of which lung adenocarcinoma (LUAD) and lung squamous cell carcinoma (LSCC) are the most common subtypes [56, 57]. Until 2004, all NSCLC subtypes—LUAD, LSCC, and large cell carcinoma—were treated in the same manner with chemotherapy (cisplatin or carboplatin in combination with either docetaxel, paclitaxel, gemcitabine, or vinorelbine). The FDA approval of gefitinib—the first EGFR inhibitor—was a game changer in NSCLC treatment, leading to stratification of patients with activating *EGFR* mutations to targeted therapy [58].

Lung cancer treatment is stage‐specific. Early‐stage disease can be cured by surgical resection, while locally advanced disease demands multimodal treatment, including chemotherapy, radiotherapy, and surgery for chosen cases. Intrinsically, due to the usually late prognosis, NSCLC is a metastatic disease (henceforth, stage IV or metastatic is applied to NSCLC or LUAD) with gloomy prognosis. Its response rate is about 30%, progression‐free survival (PFS) 4–6 months, and median overall survival (OS) about 12 months; however, toxicity usually applies to all treatment strategies. In the past, some physicians withheld chemotherapy on the basis of patients’ age (with the cutoff arbitrarily set at 70 years) [57].

### 3.1 Early detection

Although the understanding of lung cancer pathobiology has significantly improved over the last few decades, poor disease prognosis is partially attributed to late stages at diagnosis, given that there are very few early symptoms [59]. When diagnosed at an early stage, patients with NSCLC have a 5‐year survival rate of about 71%. For patients diagnosed with stage IV disease, it is less than 2% [60]. Early diagnosis may thus improve patient outcome [61], especially if ctDNA‐guided adjuvant therapy administration reaches the bedside, as proposed in the TRACERx consortium study [62, 63]. Therefore, there is still a considerable need to develop noninvasive integrative biomarkers for early detection of lung cancer.

Despite the fact that several alterations have been noted in histologically normal bronchial epithelium specimens of smokers [64], early diagnosis in NSCLC remains low. Molecular changes are detected in hyperplasia and dysplasia, stages that precede carcinoma in situ and invasive carcinoma [57]. Recent studies show that aneuploidy and driver mutations also precede cancer diagnosis by several years [65]. Driver fusion oncogenes in LUAD could arise in the early decades of life, with a long latency period before diagnosis [66].

In 2018, Cohen *et al*. [67] developed CancerSEEK—a multianalyte test that can detect eight human cancer types through determination of the levels of circulating proteins and mutations in ctDNA. For lung cancer, the probability of detection reached 75%. It seems that ctDNA combined with protein biomarkers might serve as an opportunity to detect cancers before they metastasize, when it is not yet evident radiologically. At this stage, patients can be cured in up to 50% of cases with systemic therapies. Nonetheless, further studies should be performed for the possible implementation of CancerSEEK in the clinical practice.

**Fig. 1:**
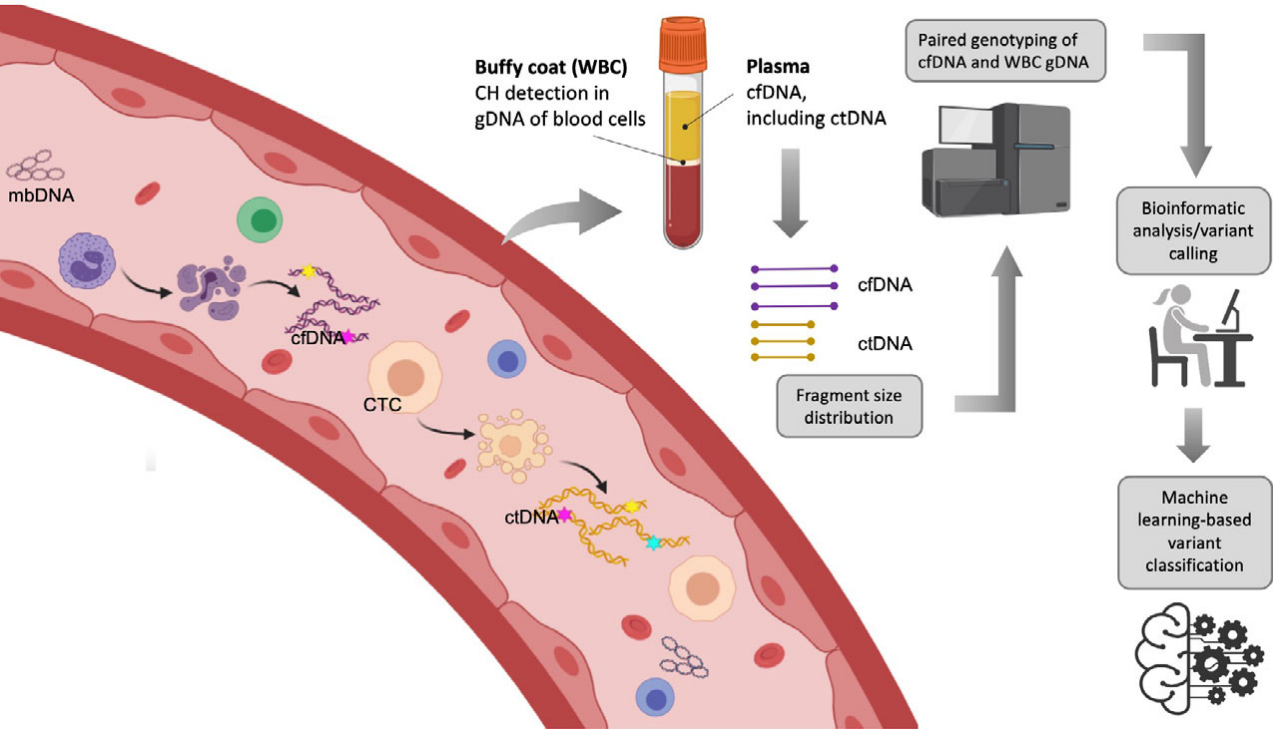
Strategies to filter clonal hematopoiesis (CH) from tumor‐derived mutated circulating free DNA (cfDNA). Plasma‐derived cfDNA, including circulating tumor DNA (ctDNA), is subjected to fragment size analysis to better discriminate between tumor‐ and non‐tumor‐derived cfDNA. Then, along with white blood cell (WBC)‐derived genomic DNA (gDNA), cfDNA is sequenced and analyzed via a rigorous bioinformatic pipeline. CH may be excluded by filtering nonsynonymous mutations except for the positive selection analysis, mutational signature analysis, and genes canonically associated with CH [48].

As the majority of cfDNA originates from hematopoietic cells, these CH mutations are detected in plasma and, without appropriate controls, can be incorrectly attributed to tumor. High‐sensitivity cfDNA analyses have identified CH mutations in 60–90% of individuals without cancer and shown them to be age‐related [43, 44]. To avoid calling these false‐positive CH mutations, white blood cell controls, fragment length discrimination, CH‐associated variant filtering, and deep‐error controlled sequencing are required (Fig. 1).

To address this issue, in their latest discovery, Chabon *et al*. [48] implemented certain improvements to cancer profiling by deep sequencing (CAPP‐Seq) [68] of ctDNA analysis at lung cancer screening. Although very low levels of ctDNA were detected in early‐stage lung cancer patients, its presence was confirmed as strongly prognostic. The mutational profile of total cfDNA from lung cancer patients and risk‐matched controls revealed nonrecurrent CH, and the mutations were detected on longer cfDNA fragments. A machine‐learning method called ‘lung cancer likelihood in plasma’ (Lung‐CLiP) allowed for robust discrimination of early‐stage lung cancer patients from risk‐matched controls [48]. These findings give hope for employment of ctDNA‐based screening methods into the clinical practice, hence reducing cancer‐related mortality by increasing the early‐detection rate.

### 3.2 Personalized treatment for NSCLC

Targetable oncogenic drivers account for almost 25% of LUADs, of which *EGFR* mutations are the most frequent [69]. The use of targeted therapies has reduced lung cancer mortality [70]. Based on Surveillance, Epidemiology and End Results (SEER) cancer registries, among men, incidence‐based mortality from NSCLC decreased by 6.3% annually from 2013 through 2016. Similar patterns were found among women with NSCLC [70].

The Lung Cancer Mutation Consortium (LCMC) performed the first large‐scale study in LUADs for detection of oncogenic driver mutations in the United States. Multiplex genotyping for mutation detection was performed in several sites, using any of three methods: matrix‐assisted laser desorption/ionization time‐of‐flight mass spectrometry (Sequenom, Arizona Research Laboratories), multiplex single‐nucleotide extension sequencing (SNaPshot, Applied Biosystems), or Sanger sequencing with peptide nucleic acid probes [71]. The LCMC prioritized genotyping *EGFR*, *KRAS*, erb‐b2 receptor tyrosine kinase 2 (*ERBB2*), AKT serine/threonine kinase 1 (*AKT1*), B‐Raf proto‐oncogene (*BRAF*), serine/threonine protein kinase MEK1 (*MEK1*), *NRAS*, *PIK3CA*, ALK receptor tyrosine kinase (*ALK*), and MET proto‐oncogene (*MET*). *KRAS* mutations were the most frequent, found in 182 of 733 specimens (25%), followed by sensitizing *EGFR* mutations (exon 19 deletions, L858R, L861Q, and G719X) in 122 of 733 specimens (17%). This kind of *EGFR* mutation results in sensitivity to tyrosine kinase inhibitors (TKIs). *ALK* rearrangements occurred in 57 of 733 specimens (8%). The median survival of patients with each of the five most common oncogenic drivers ranged from 2.0 years (mutations in two genes) to 4.3 years (*ALK‐*rearranged tumors). The 260 patients with an oncogenic driver and treatment with a targeted drug had a median survival of 3.5 years; the 318 patients with a driver and no targeted therapy had 2.4 years median survival; and the 360 patients with no driver identified had 2.1 years (*P* < 0.001) [71].

Other nationwide studies were performed following the LCMC study [71], including a genomic screening network (LC‐SCRUM‐Japan) and the French nationwide IFCT‐InCa project, as well as a project in Cologne, Germany (reviewed in ref. [69]). All these initiatives paved the way for the current genomic classification of LUAD and set the basis for the genomic classification of LSCC [69, 72].

Recently, the TARGET study used a ctDNA assay involving a panel of 641 cancer‐associated genes. For the first 100 patients, ctDNA data showed good concordance with tissue (78% concordance) and identified potentially actionable mutations in 41% of patients. Of these patients, 11 of 41 (27%) went on to a matched therapy [73].

#### 3.2.1 *EGFR*‐mutant LUAD genomic assessment

Targeted next‐generation sequencing in tumor tissue from metastatic NSCLC is gradually becoming more used for the identification of targetable driver mutations and gene fusions. However, the first question that comes up when a driver actionable mutation is detected is: What is the meaning of the other, often co‐occurring, mutations and gene alterations reported?

*EGFR*‐mutant NSCLC was analyzed using the MSK‐IMPACT assay, a clinical test that detects mutations, copy‐number alterations, and select fusions involving 341 (version 1), 410 (version 2), or 468 (version 3) cancer‐associated genes [74]. The median number of co‐mutations was 5 (range 0–19) in samples prior to EGFR TKI therapy. The most frequent co‐occurring mutations were tumor protein p53 (*TP53*) (60% *n* = 119), *PIK3CA* (12%, *n* = 23), catenin beta 1 (*CTNNB1*) (9%, *n* = 18), and RB transcriptional corepressor 1 (*RB1*) (10%, *n* = 19). The most frequent concurrent amplifications were *EGFR* (22%, *n* = 45), NK2 homeobox 1/transcription termination factor 1 (*NKX2‐1/TTF‐1*) (15%, *n* = 29), MDM2 proto‐oncogene (*MDM2*) (12%, *n* = 23), cyclin‐dependent kinase 4 (*CDK4*) (10%, *n* = 21), and forkhead box A1 (*FOXA1*) (10%, *n* = 20) [74]. FOXA1 is a transcription factor that is frequently mutated in prostate, breast, bladder, and salivary gland tumors [75]. The median PFS was 11 months; however, if the *TP53* mutation was also present, then the median PFS was reduced (6 months) [74]. PFS was 5 months when pretreatment *MET* amplification (2%, *n* = 4) was noted. The presence of *TP53* alterations was also associated with shorter survival [74]. The *TP53* concurrent mutation also predicted shorter PFS to EGFR TKI in *EGFR*‐mutated NSCLC [76]. More recent studies [77] reconfirm the Helena Yu co‐mutation plot of genomic alterations [74] and help to gain insights on co‐acquired alterations through the evolution of TKI therapy in NSCLC patients [77]. *PIK3CA* mutations showed a domain‐dependent effect on PFS. Mutations in the kinase domain (Y1021H and H1047R), helical domain (E542K), and C2 domain (N345K) were associated with poorer PFS, while mutations in the p85‐binding domain (R88Q, R108H, and K111E) were associated with an improved survival [77]. Intriguingly, multiple driver mutations occur in the same gene, especially in *PIK3CA* (10% of samples) and *EGFR* (10%) [78]. In fact, The Cancer Genome Atlas (TCGA) research network showed that the principal driver alterations in NSCLC (i.e., LUAD)—either *EGFR* mutations or *KRAS* mutations—include the co‐occurrence of several others, commonly with *TP53* mutations, as well as serine/threonine kinase 11 (*STK11*) and kelch‐like ECH‐associated protein 1 (*KEAP1*) mutations and alterations [79].

#### 3.2.2 Detection of *EGFR* mutations in ctDNA

In our group [80], *EGFR* mutations in ctDNA were assessed in a large cohort of 1026 NSCLC patients, and sensitizing *EGFR* mutations were found in 113 patients (11%). More than 50% of samples had <10 pg mutated genomes per µL, with allelic fractions below 0.25%. Patients treated first line with TKI had an objective response rate of 72% and a median PFS of 11 months. Of the 105 patients screened after progression to EGFR TKIs, sensitizing mutations were found in 56%, and the acquired T790M mutation was found in 35%. Detection of *EGFR* mutations in plasma ctDNA was used as a selection criterion for first‐line gefitinib in patients with LUAD (BENEFIT study) [81]. The objective response rate among 188 patients was 72%, while median PFS was 9.5 months. Of 167 patients with available blood samples, 147 (88%) had clearance of *EGFR* mutations in ctDNA at week 8, and median PFS was longer for these patients than for the 20 patients whose *EGFR* mutations persisted at week 8 (11 months vs 2.1 months, *P* < 0.0001). From baseline next‐generation sequencing (NGS) data in 179 patients [using an ultra‐deep (20 000×) 168‐gene panel named LungPlasma (Burning Rock Biotech, Guangzhou, China)], three subgroups were seen: those with *EGFR* mutations alone (*n* = 58), those with mutations in *EGFR* and tumor‐suppressor genes [*TP53* (65%), *RB1* (8%) or phosphatase and tensin homolog (*PTEN*), (3%) (*n* = 97)], and those with mutations in *EGFR* and oncogenes [*MET*, *ERBB2*, *KRAS*, *BRAF*, ret proto‐oncogene (*RET*) or ROS proto‐oncogene 1 (*ROS1*) (*n* = 24)]. Corresponding median PFS in these subgroups was 13.2 months, 9.3 months, and 4.7 months, respectively [81].

An earlier seminal study deciphered the evolution and clinical impact of co‐occurring genetic alterations in 1122 *EGFR*‐mutated NSCLC patients by means of the Guardant 360 ctDNA assay, which covered single‐nucleotide variants, small insertions/deletions (indels), gene rearrangements/fusions, and copy‐number gains across 68 clinically relevant cancer genes [82]. *PIK3CA* and *CTNNB1* mutations were noted in tumor tissue, as described above [74]. Interestingly, concurrent genomic alterations detectable in *EGFR*‐mutant NSCLC patients encoding the *EGFR* C797S mutation were also found [82]. Using new versions of the Guardant360 ctDNA assay, covering 73 cancer‐related genes, two studies have reported great concordance between the ctDNA assay and tissue‐based clinical genotyping. In the United States, 282 patients were evaluated in the NILE study (Non‐Invasive versus Invasive Lung Evaluation) [83]. In tissue‐positive patients, the biomarker was identified alone (12/60) or concordant with ctDNA (48/60), with an 80% ctDNA sensitivity. Importantly, the ctDNA median turnaround time was faster than tissue (9 vs 15 days; *P* < 0.0001) [83]. We conducted a similar prospective study in Spain [84] in 186 NSCLC patients, confirming that the Guardant360 NGS ctDNA assay was not inferior to standard‐of‐care tissue testing in detecting recommended biomarkers, further confirming that ctDNA‐based first‐line therapy produces outcomes similar to tissue‐based testing [84].

As confirmed in multiple studies, detection of *EGFR* mutations in plasma serves as a satisfactory surrogate for tissue biopsy. Significant weight should be put on the co‐occurring genetic alterations, since their detection serves as an important prognostic marker.

#### 3.2.3 Significance of *EGFR* L858R mutation in ctDNA

We carried out a large‐scale ctDNA screening of patients with NSCLC for *EGFR* mutations in Spain. *EGFR* mutations were found in 350 of 2015 patients (16.6%). For 217 patients who received erlotinib, the adjusted hazard ratios for the duration of PFS were 2.94 for men (*P* < 0.001); 1.92 for the presence of the L858R mutation, as compared with a deletion in exon 19 (*P* = 0.02); and 1.68 for the presence of the L858R mutation in paired serum DNA, as compared with the absence of the mutation (*P* = 0.02) [85]. After examining the ctDNA of 97 patients included in the EURTAC phase 3 trial [86] using a peptide nucleic acid‐mediated 5’ nuclease real‐time polymerase chain reaction (TaqMan) assay, it was determined that median OS was shorter in patients with the L858R mutation in ctDNA than in those with the exon 19 deletion (13.7 vs 30 months, *P* < 0.01). Moreover, univariate analysis of patients with *EGFR* mutations in ctDNA identified the L858R mutation in tumor tissue or in ctDNA as a marker of shorter OS (hazard ratio, 2.70, *P* < 0.001) and PFS (hazard ratio, 2.04, *P* = 0.008). For patients with the L858R mutation detected primarily in tissue, median OS was 13.7 months for patients with the L858R mutation in ctDNA and 27.7 months for those with no mutation detected (*P* = 0.03). The conclusion was that the L858R mutation in ctDNA might be a novel surrogate prognostic marker [87].

The FLAURA phase 3 trial of osimertinib vs gefitinib in first line also showed a shorter PFS and OS in *EGFR*‐mutant NSCLC harboring the L858R mutation in tumor tissue [88, 89]. Anterior gradient 2 (*AGR2*), a disulfide isomerase that promotes lung tumorigenesis, is significantly expressed (using TCGA datasets) in *EGFR* L858R airway tumors induced by transforming growth factor alpha (TGFA; an EGFR ligand), but not in normal lung. Experimental data points out that AGR2 may contribute to the growth of *EGFR* L858R airway lung tumors induced by TGFA. In addition, TGFA induces the expression of *AGR2* in human *EGFR*‐mutant LUAD. As explained below, TGFA‐mediated fibrosis associated with *EGFR‐*mutant lung tumors *in vivo* may induce growth factors (e.g., FGF) that confer resistance to EGFR TKIs, gefitinib, erlotinib, afatinib, and osimertinib. It has been posited that TGFA is a therapeutic target for recurrent *EGFR*‐mutant lung cancer (e.g., osimertinib‐resistant LUAD) [90]. Although the biological background of L858R mutations has not been elucidated, it is remarkable that multiple driver mutations in the same *EGFR* gene can occur for L858R, in contrast with the *EGFR* exon 19 deletion [91]. A number of rare (minor) mutations are found in extracellular and transmembrane domains, together with the L858R major driver mutation. These findings lead us to speculate that multiple mutations in the same oncogene cooperate to potentiate its tumor‐promoting activity [90].

#### 3.2.4 Landscape of acquired *EGFR* mutations, other mutations, and gene fusions in *EGFR*‐mutant NSCLC

*EGFR*‐mutant NSCLC patients treated with TKI often develop acquired *EGFR* mutations that, until now, were only examined in re‐biopsies. As an example, a patient that harbored *EGFR* exon 19 deletion was treated with afatinib [92]. After progression, the second biopsy still revealed the presence of *EGFR* exon 19 deletion and the acquired *EGFR* exon 20 T790M. Treatment was then switched to osimertinib (a mutant‐specific third‐generation EGFR TKI). When the third biopsy was performed, *EGFR* exon 19 deletion was pervasively identified, with disappearance of *EGFR* exon 20 T790M and emergent *EGFR* exon 20 C797S (acquired with osimertinib). The patient was treated with gefitinib (EGFR inhibitor, first generation) which induced tumor response once more [92].

Previously, it was suggested that combining first‐ and third‐generation TKIs in first‐line therapy could be crucial, because neither a T790M, nor a C797S mutation, alone, would be able to drive resistance to the combination [93]. For T790M‐positive, erlotinib‐resistant NSCLCs that develop a C797S mutation following therapy with a third‐generation TKI, the configuration of the T790M and C797S mutations influences how the cells can respond to therapy. If the two mutations are in *trans* (on separate alleles), then the combination of first‐ and third‐generation TKIs can restore EGFR inhibition. Conversely, if the two mutations are in *cis* (on the same allele), the cells are refractory to any of the EGFR TKIs, as well as the combination of first‐ and third‐generation inhibitors [93]. It was foreseen that clinical assessment of the *cis* versus *trans* configuration can be examined by NGS, since T790M and C797S mutations are in close enough proximity to coexist on a significant number of individual sequencing reads [93, 94]. Interestingly, it was shown that, at a concentration of 1 µmol·L^−1^, afatinib can inhibit mutant *EGFR* with C797S in the absence of T790M [93].

Osimertinib is the preferred first‐line therapy for *EGFR*‐mutant NSCLC [88, 89]; however, resistance unavoidably develops in patients. Resistance is mediated by acquired secondary mutations in *EGFR*. In addition to C797S, others also occur, such as L718Q [95]. Analysis of ctDNA data from patients disclosed that L718Q mutations usually appear in the context of an L858R driver mutation [96]. This adds evidence to the observation that additional mutations occur in the presence of *EGFR* L858R, rather than in *EGFR* exon 19 deletion [78].

On the same lines as Niederst’s observations [93], treatment in mice revealed that both erlotinib and afatinib caused regression of osimertinib‐resistant C797S‐containing tumors, whereas only afatinib was effective in L718Q mutant tumors. Combination of first‐line osimertinib plus erlotinib could prevent the emergence of secondary mutations in *EGFR* [96].

A novel *EGFR* G724S mutation, causing resistance to osimertinib, occurs with exon 19 deletion, but not L858R. In addition, the exon 19 deletion/G724S retains sensitivity to afatinib, but not to erlotinib [97]. *EGFR* C724S was identified in 4% of postosimertinib patients treated with first‐line osimertinib, whereas C797X was identified in 29% of postosimertinib patients treated with later‐line osimertinib [98].

Targeted NGS for 416 cancer‐related genes was carried out in 93 osimertinib‐resistant NSCLC patient samples, mainly in ctDNA, and matched pretreatment samples of 12 patients. A co‐mutation plot of postosimertinib‐treated patients revealed two subgroups of patients: those with major *EGFR* tertiary mutations at the positions of L718/G719, L792, and G796/C797 (identified with a frequency of 9.7%, 10.8%, and 24.7%, respectively). In most cases, mutations were also noted in *EGFR* T790M and *TP53* [99]. Almost all patients without *EGFR* resistance mutations showed *TP53*, *MET*, *KRAS*, or *PIK3CA* mutations [99].

Median OS after osimertinib progression (osimertinib given as second‐line in *EGFR* T790M mutant NSCLC patents) was 5.4 months in 40 patients from the AURA study, whose plasma was available after disease progression [100]. Twelve (30%) of these had the T790M mutation (four of whom also had C797S). Patients without detectable *EGFR*‐activating mutations in plasma before treatment had the best overall and postprogression survival (22.4 months and 10.8 months, respectively). Loss of T790M but presence of *EGFR*‐activating mutations in plasma was associated with the shortest PFS (median 2.6 months). Importantly, in 22 postprogression tumor samples, one squamous cell and two small‐cell transformations were seen. In addition, the number of patients was small and T790M was found in 50% of samples, C797S in 17%, *MET* amplification in 50%, *BRAF* mutations in 8%, and *KRAS* mutations in 8%.

Once we have the data from a ctDNA analysis, what should we do with it? What do we do when facing the multiplicity of genomic aberrations described? Is it a basic rule in medicine to bear in mind their frequency? Secondly, are the acquired driver co‐alterations druggable? A recent study has shed light on these issues [98]. MSK‐Fusion Solid, a custom RNAseq panel, was used to detect fusions in cases where no resistance mechanism was identified by NGS and sufficient tissue was available. Among 62 patients, histological squamous transformation was identified in 15% of first‐line osimertinib cases and 14% of later‐line cases. Nineteen percent of patients treated with first‐line osimertinib had off‐target genetic resistance (2 *MET* amplifications, 1 *KRAS* mutation, 1 *RET* fusion, and 1 *BRAF* fusion), whereas 4% had an acquired *EGFR* mutation (*EGFR* G724S). Patients with squamous transformation acquired *PIK3CA* mutation, chromosome 3q amplification, and fibroblastic growth factor (*FGF*) amplification. The compound mutation *EGFR* S768 + V769L and the mutation *MET* H1094Y were also identified. Longitudinal analysis of two patients, who received later‐line osimertinib with emergence of *ALK* fusion, revealed that one patient acquired an *EGFR* C797S mutation and lost the *ALK* fusion after treatment with osimertinib and alectinib. The other patient acquired the *ALK* G1202R mutation after treatment with osimertinib and alectinib [98]. Recent studies confirm the validity of ctDNA analysis for response assessment to osimertinib [101] and others regarding the prognostic impact of *TP53* mutations, suggesting that *EGFR*‐mutant and *TP53* wild‐type patients may benefit from the combination of EGFR TKI with bevacizumab [102].

Great progress has also been made in the use of ALK inhibitors for the treatment of patients with *ALK*‐positive NSCLC, from crizotinib to second‐generation ALK inhibitors, including alectinib, brigatinib and ensartinib, and lorlatinib, a third‐generation ALK inhibitor [103]. The combination of ALK and MET inhibitors is emerging as a plausible efficient combination, since *MET* amplification has been detected in 15% of tumor biopsies from patients relapsing on next‐generation ALK inhibitors, including 12% and 22% of biopsies from patients progressing on second‐generation inhibitors or lorlatinib, respectively [104]. Also, two tumor specimens harbored suppression of tumorigenicity 7 (*ST7*)‐*MET* rearrangement [105]. A recent phase 2 study, VISION, evaluates tepotinib (a MET inhibitor) for the treatment of NSCLC with *MET* exon 14 skipping mutations. PFS with tepotinib was 11 months (tissue biopsy, *n* = 60), 8.5 months (liquid biopsy, *n* = 66) and 8.5 months (combined biopsy, *n* = 60) [106]. In addition, for *MET* exon 14 skipping mutations, patients treated with crizotinib (MET inhibitor; PROFILE 1001), PFS was significantly shorter in patients with *MET* mutations detected in ctDNA versus nondetected in ctDNA, with a median of 3.6 months vs 7.8 months (hazard ratio = 2.27, *P* = 0.06) [107].

It is clear that targeted NGS in tissue and plasma provides complementary theranostic information in the current era of single targeted therapy. However, the genomic evaluation could become complex with the cornucopia of genomic alterations that are being developed, and tracking plasma is the most suitable advance that the practitioner has, although it poses multiple hurdles on how to treat the patient. One appealing case described an *EGFR* L858R patient that also displayed *EGFR* G719S in four different regions of the resected lung tumor, in different allelic proportions, as well as SWI/SNF‐related, matrix‐associated, actin‐dependent regulator of chromatin, subfamily a, member 4 (*SMARCA4*) [108]. At the time of relapse, she received afatinib and a liver metastasis biopsy was performed where L858R was found in an allelic fraction of 14%, together with other gene mutations. After 2 years of afatinib, a resection of an ovarian metastasis revealed mutation of the *EGFR* gene in six different regions: L858R, G719S (allelic fraction range, 40–50%), and *EGFR* C797S (allelic fraction range 9–14%) in five of the six areas of the ovary. *SMARCA4* mutation was found in all six ovarian areas analyzed, with an allelic fraction of more than 40%. After the ovary metastasis resection, the patient continued afatinib for more than 2 years without recurrence [108]. Almonertinib is a third‐generation EGFR TKI with high selectivity for *EGFR*‐sensitizing and T790M resistance mutations that show great inhibitory activity against T790M, T790M/L858R, and T790M/Del19 [109]. The secondary objective of a recently opened multicenter, open‐phase II clinical study (NCT04841811), in patients with unresectable stage III NSCLC is to assess the safety of different treatment decisions guided by ctDNA monitoring [110].

Targeted NGS ctDNA assays are warranted to manage the complexity of genomic alterations; however, intrinsic and acquired resistance also involves the identification of pathways that influence sensitivity, such as in *KRAS* G12C‐mutant NSCLC, where the combination of the KRAS G12C inhibitor, PI3K inhibitor, and SHP2 inhibitor caused tumor regression in mouse models with acquired resistance to AMG510 [111]. Co‐treatment in *EGFR‐*mutant NSCLC is highly recommended to improve PFS and OS. We and other groups have shown that targeting STAT3, YAP1, and SHP2 could have great synergism *in vitro* and *in vivo* in *EGFR*‐mutant cell line models [112, 113, 114] and that, by preventing NF‐κB signaling activation, the formation of acquired *EGFR* mutations could be prohibited [115].

#### 3.2.5 ctDNA as a biomarker for immunotherapy

Most recently, the introduction of immunotherapy with promising responses in subgroups of cancer patients preceded the search for biomarkers to better stratify patients. This raised the question of whether studying a tumor microenvironment from the tissue biopsy was crucial for predictive biomarker discovery or whether a biomarker could be reasonably sought in a peripheral blood sample. It has been demonstrated that the latter may be achieved via analysis of tumor mutation burden (TMB) in ctDNA, adding another liquid biopsy biomarker to a constantly expanding list of blood tests for the management of cancer. An interesting approach to combine two liquid biopsy biosources would be to complement TMB detected on ctDNA with PD‐L1 assessment on CTCs [116]. The recently opened BESPOKE clinical trial (NCT04761783) is to examine the impact of SIGNATERA™ (a personalized and tumor‐informed 16‐plex NGS assay to detect ctDNA) on clinical decision‐making regarding immunotherapy for treatment of solid tumors [117].

Interestingly, fusion‐driven LUADs often have *SETD2* mutations, which are not seen in LUADs with *EGFR*, *KRAS*, *BRAF*, or *MET* mutations [66]. This finding is of interest, since *ALK* or *ROS1* fusion‐driven LUADs show poor benefit with immune checkpoint inhibitors (anti‐PD‐1 or anti‐PD‐L1 monoclonal antibodies) [118, 119]. SETD2 methyltransferase mediates STAT1 methylation on lysine 525, being an essential signaling event for interferon‐alpha‐dependent antiviral immunity [120]. The current management of metastatic NSCLC has evolved from chemotherapy to chemotherapy plus immune checkpoint inhibitors in a large number of NSCLC patients with no identifiable driver oncogene mutations or fusions [56, 121].

Despite the progress in immunotherapy, not all NSCLC patients respond. Whole‐exome sequencing of 104 patients treated with immune checkpoint inhibitors identified that corrected TMB (cTMB) adjusted for tumor purity predicted the benefit of immunotherapy, as well as smoke‐related mutational signature and human leukocyte antigen status. However, mutations in receptor tyrosine kinase (RTK) genes were indicative of no response [122].

## 4 Conclusions and perspectives

In the hope of overcoming several obstacles and challenges resulting from classical tissue biopsy‐based diagnosis, liquid biopsy emerged as a robust tool for ctDNA monitoring and disease detection and monitoring. Importantly, liquid biopsies trump tissue biopsies when there is insufficient material for testing or its quality is unsatisfactory. In addition, a patient’s poor performance status and tumor accessibility are often a substantial concern.

**Fig. 2:**
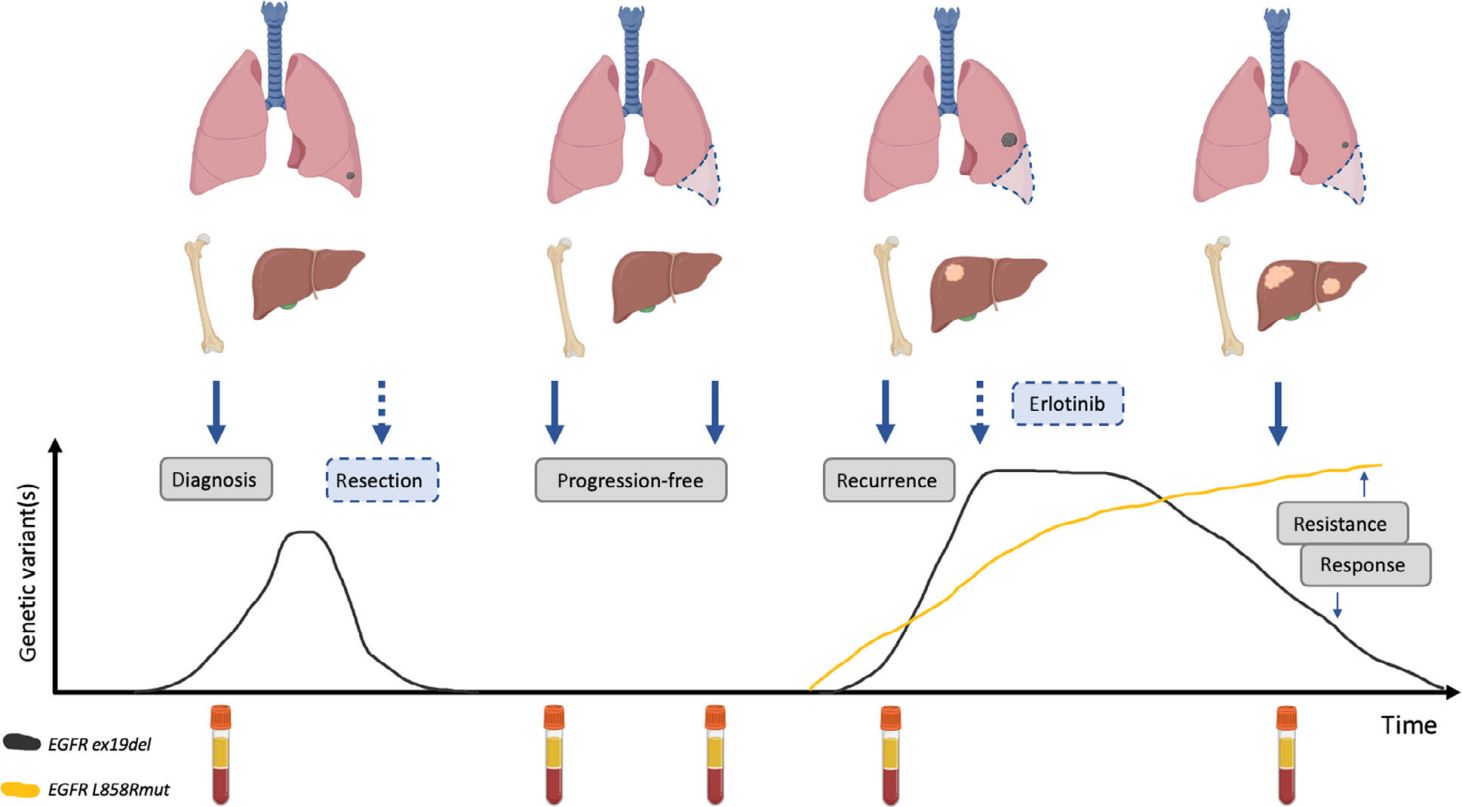
ctDNA‐based liquid biopsy in clinical use. Lung cancer was diagnosed at early stage, *EGFR* exon 19 deletion (ex19del) was detected, and the tumor was subjected to resection. Subsequently, two liquid biopsies detected no mutated circulating tumor DNA (ctDNA), indicating no progression of the disease. At the recurrence, *EGFR* ex19del and L858R mutation (L858Rmut) was detected in ctDNA and erlotinib was administered to the patient. L858R mutant cells metastasized to the liver and developed resistance to erlotinib (yellow line). Tumor derived from the *EGFR* ex19del clone responded well to the therapy (gray line). Solid arrows indicate liquid biopsy sample collection time points; dashed arrows indicate treatment procedures.

Tumors are highly heterogeneous, and sampling in its entirety is challenging: How well does a small tissue biopsy sample represent the whole tumor? In patients with multiple metastases, to gain a holistic view of the disease biopsy, samples should be collected from all of the (known) metastatic sites (Fig. 2). Since the blood reaches most tumor sites in patients with advanced cancers, it is perhaps reasonable to speculate that blood‐based liquid biopsies might better reflect tumor heterogeneity. Moreover, tumors evolve over time and can modify their molecular fingerprint, making clinical decisions based on historical biopsy data insignificant. The limitation of acquiring tissue biopsy samples longitudinally to determine disease response or monitor relapse is also reduced by the liquid biopsy approach, since multiple samples can be collected noninvasively over time (Fig. 2).

Although early‐detection strategies based on ctDNA are promising, numerous hindrances must be addressed before they can be robustly applied in the clinic. False‐positive results can be precarious for any screening assay. Experience thus far suggests that benign tumors and non‐neoplastic conditions do not generally give rise to ctDNA [29], so the ‘overdiagnosis’ of benign tumors is not likely to pose a major problem. Moreover, strategies to filter out CH signal are being developed (Fig. 1) [34, 35, 36].

ctDNA can be robustly detected in plasma when a significant number of copies of mutant ctDNA are shed into blood. However, when the amounts of ctDNA are too low, analysis of individual mutant loci might result in a negative result because of sampling background noise even when using an assay with perfect analytical sensitivity [123]. It is much easier to detect a single mutation in the follow‐up of an advanced disease and more demanding in the early stage where higher depth for detecting a broad panel of mutations requires more ctDNA input. Where the amount ctDNA isolated is not sufficient for an NGS analysis, alternative platforms, such as NanoString nCounter, can be of service [124].

A revolutionary approach for cancer management was proposed by Wan and colleagues [125], in which tumor‐guided personalized ctDNA screening is performed in longitudinally collected plasma samples. Such patient‐specific mutation lists provide an opportunity for highly sensitive monitoring from a range of sequencing data types using methods for signal aggregation, weighting, and error suppression [125]. All this makes ctDNA still the most useful technique for companion diagnostics approaches where the mutation is druggable.

Despite its potential, ctDNA analysis is not suitable to diagnose all cancers, since some tumor types (e.g., gliomas and sarcomas) are poor ctDNA shedders. Moreover, ctDNA‐based assays are applicable in tumors with higher TMB, whereas, for example, glioblastoma multiforme or pancreatic adenocarcinoma are barely detectable in blood, especially at earlier stages, due to their low shedding and TMB. Thus, Poore *et al*. [17] have approached this limitation: They addressed the possibility of using mbDNA to discriminate between cancer and healthy patients. In short, their findings suggest that mbDNA may serve as a biomarker for cancer diagnosis and detection even for low‐TMB cancers. This new path in liquid biopsy cancer diagnostics has been opened by highlighting another clinical application of microbiome‐based assays [17, 126].

In conclusion, ctDNA‐based liquid biopsies can be a powerful tool for cancer diagnosis, monitoring, prognosis, and individualized treatment and can completely change the current paradigms of cancer management, especially in a multianalyte setting. However, considerable research and development are still needed to improve the isolation, enrichment, and downstream analysis of all circulating biomarkers. In our view, in current clinical practice, ctDNA measurements need to be combined with standard‐of‐care approaches and ideally combined with other blood‐based biosources.

## Conflict of interest

The authors declare no conflict of interest.
